# Do primary care quality improvement frameworks consider equity?

**DOI:** 10.1136/bmjoq-2024-002839

**Published:** 2024-07-24

**Authors:** Alice Macdonald Barrell, Lucy Johnson, Amy Dehn Lunn, John Alexander Ford

**Affiliations:** 1 University of Cambridge School of Clinical Medicine, Cambridge, UK; 2 Queen Mary University of London Wolfson Institute of Population Health, London, UK

**Keywords:** Quality improvement, Quality improvement methodologies, Health Equity, Primary care

## Abstract

**Background:**

Quality improvement (QI) is used by healthcare organisations internationally to improve care. Unless QI explicitly addresses equity, projects that aim to improve care may exacerbate health and care inequalities for disadvantaged groups. There are several QI frameworks used in primary care, but we do not know the extent to which they consider equity. This work aimed to investigate whether primary care QI frameworks consider equity.

**Methods:**

We conducted a search of MEDLINE, EMBASE and key websites to compile a list of the QI frameworks used in primary care. This list was refined by an expert panel. Guidance documents for each of the QI frameworks were identified from national websites or QI organisations. We undertook a document analysis of the guidance using NVivo.

**Results:**

We analysed 15 guidance documents. We identified the following themes: (1) there was a limited discussion of equity or targeted QI for disadvantaged groups in the documents, (2) there were indirect considerations of inequalities via patient involvement or targeting QI to patient demographics and (3) there was a greater focus on efficiency than equity in the documents.

**Conclusion:**

There is limited consideration of equity in QI frameworks used in primary care. Where equity is discussed, it is implicit and open to interpretation. This research demonstrates a need for frameworks to be revised with an explicit equity focus to ensure the distribution of benefits from QI is equitable.

**WHAT IS ALREADY KNOWN ON THIS TOPIC**

- Previous research has shown that quality improvement (QI) projects may maintain, improve or worsen underlying health inequality. Several frameworks are used to guide the implementation of QI in primary care, but the extent to which these frameworks consider equity is not known.

**WHAT THIS STUDY ADDS**

- QI frameworks in primary care lack equity considerations and tend to indirectly consider inequalities with a focus on efficiency rather than equity.

**HOW THIS STUDY MIGHT AFFECT RESEARCH, PRACTICE OR POLICY**

- Existing QI frameworks need revision to better consider equity to ensure that the benefits of QI are distributed equally.

## Background

 Quality is defined by the Institute of Medicine (IoM) as being safe, effective, patient-centred, timely, efficient and equitable.^1^ Health equity, defined as ‘the absence of unfair, avoidable or remediable differences among groups of people,’^2^ is often not considered in quality improvement (QI). It has been described as the forgotten aim of healthcare improvement.^3^

QI initiatives seek to enhance patients’ experience of healthcare and improve outcomes. In the UK, QI is incentivised in primary care nationally via the Quality and Outcomes Framework.^4^ QI is more important than ever due to worsening patient satisfaction and widening inequalities in primary care. According to the general practitioner (GP) patient survey, patient satisfaction fell from 83% in 2021 to 72.4% in 2022.^5^ We define primary care as the usual first point of contact for healthcare and management of long-term conditions, including GP services.

QI projects may maintain, improve or worsen underlying health inequalities.^6^ Without explicitly considering equity in the design and analysis of QI projects, it is easy for a project to be deemed a success while inequalities persist or worsen beneath the surface. Several examples of this exist in the published literature.^7^

A plethora of frameworks exist to support QI in primary care. Notable examples include the Royal College of General Practice (RCGP) QI wheel and the Model for Improvement.^8 9^ The extent to which these models consider equity has not previously been formally assessed. There have been several calls for QI to be more equity focused,^10 11^ but if the existing frameworks do not include equity considerations, it is likely that these calls will go unheard. Here, we aim to explore the extent to which commonly used QI frameworks in primary care consider equity.

## Methods

We undertook a document analysis of commonly used QI frameworks in primary care.

First, we identified a long list of potential frameworks by mapping of the published literature and a targeted search of key websites. We defined a QI framework as a proposed methodology for the design, testing and implementation of a project to improve healthcare quality.

Literature mapping was undertaken through a search of MEDLINE and EMBASE using search terms from previous QI reviews.^12^ Key search terms were ‘quality improvement’ and ‘primary care’ or ‘general practice’. The purpose was to identify commonly used QI frameworks in primary care from the literature. We focused on articles published in the past 10 years describing a QI project undertaken in primary care in a high-income country. We excluded editorials, commentaries, or conference abstracts, and non-English language publications. A researcher (AMB) screened all the titles and abstracts identified from the electronic database search in Rayyan, an online tool to support literature reviews, identifying those which described a QI project in primary care. We then reviewed the full text, recording the framework used in addition to some high-level article characteristics (table 1). In addition, we searched the following websites; National Health Service (NHS) England, RCGP, Institute of Healthcare Improvement (IHI), Health Foundation and the Kings Fund.

**Table 1:** Characteristics of the included documents

	Number of articles
Country	
USA	85
UK	20
Australia	5
Canada	5
Other	5
Framework	
None recorded	50
PDSA	43
Model for Improvement	14
CQI	4
Always Events	3
Care bundle	3
Lean	3
Six Sigma	2
FADE	1
FOCUS-PDSA	1
PDCA	1
PRISM	1
RE-AIM	1
QIE	1
Condition/topic	
Child health	16
Diabetes	15
Screening/testing	13
Prescribing	11
Adult mental health	6
Hypertension	6
Sexually Transmitted Diseases	6
Other	47

CQIContinuous Quality ImprovementFADEFocus, Analyse, Develop, ExecuteFOCUSFind, Organise, Clarify, Understand, Select PDCAPlan, Do, Check, Act PDSAPlan, Do, Study, ActPRISMPractical, Robust, Implementation and Sustainability ModelQIEQuality Implementation and Evaluation RE-AIMReach, Efficacy, Adoption, Implementation and Maintenance

We reviewed the long list of QI frameworks identified from the key websites and literature mapping with an expert panel of primary care professionals. The aim of this was to prioritise frameworks commonly used in practice. In addition, we asked the expert panel for any frameworks which we had not already identified.

We then identified the primary guidance documents for each framework. Framework guidance documents were selected from sources such as NHS websites, IHI guidance and the published literature. Where possible, primary care-specific guidance documents were chosen.

QI framework documents were imported to NVivo V.14 and thematically coded for equity considerations. We used the WHO definition of health equity as ‘the absence of unfair, avoidable or remediable differences among groups of people, where those groups are defined socially, economically, demographically or geographically’.^2^ And, we conceptualised this further by using the PROGRESS Plus characteristics.^13^ We used inductive coding to reflect the different themes carried across the framework guidance documents. We coded sections of text with explicit mentions of equity or related factors, such as social determinants of health, socioeconomic deprivation and barriers to healthcare access. We also coded for implicit references to health inequality, such as targeting healthcare to need and patient involvement in QI projects. Codes were then aggregated into higher-level themes.

### Patient and public involvement

Patients were not directly involved in this research.

## Results

**Figure 1:**
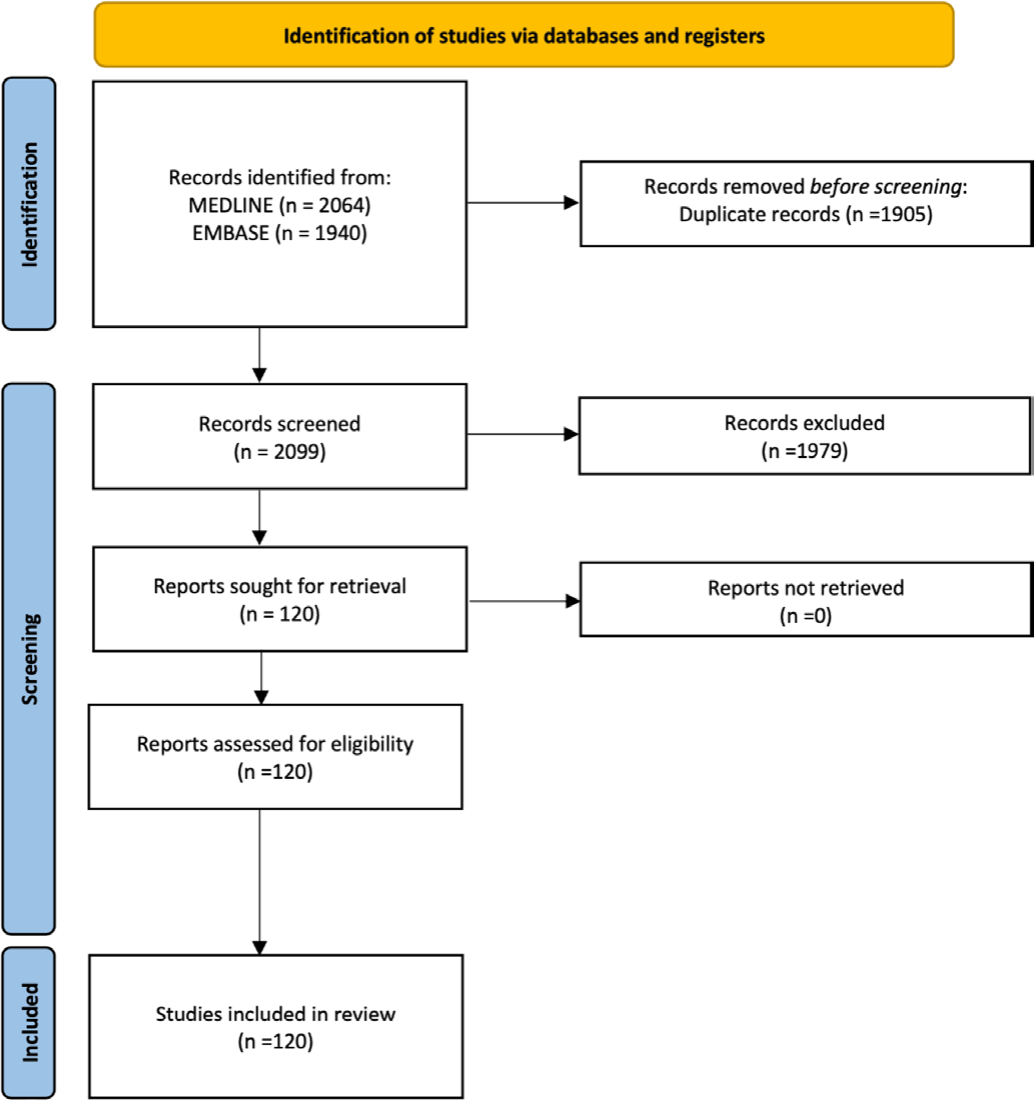
Preferred Reporting Items for Systematic Reviews and Meta-Analyses (PRISMA) flow diagram.

Our initial search found 2064 documents in Medline via Ovid and 1940 in Embase via Ovid, with 1905 removed via duplication. This left a total of 2099 documents for screening. We identified 120 articles reporting a QI project in primary care (figure 1). The majority of these projects were from the USA (n=85), with some from the UK, Australia and Canada (table 1). Many projects did not use a recognised QI framework. Of those that did, the most used framework was Plan, Do, Study, Act and the Model for Improvement. Many of the projects were based on child health and well-being, diabetes, or screening for different conditions.

We used 15 documents or websites providing guidance on the selected frameworks (table 2).

**Table 2:** Included documents

Framework	Guidance document(s)	Description
Model for Improvement	Science of Improvement: How to Improve^9^	Institute of Healthcare Improvement (IHI) webpage on The Model for Improvement
Plan, Do, Study, Act (PDSA)	Quality Improvement Essentials Toolkit^31^	IHI worksheet to guide the implementation of PDSA.
	PDSA cycles and the Model for Improvement^32^	NHS England & NHS Improvement publication outlining use of the framework in the NHS.
RCGP QI Wheel for Primary Care	Quality Improvement for General Practice^8^	Royal College of General Practitioners (RCGP) official publication detailing their recommended QI framework.
Care bundles	Using Care Bundles to Improve Healthcare Quality^33^	IHI white paper on this framework.
Lean	Going Lean in the NHS^18^	Publication by NHS Institute for Innovation and Improvement
	Going Lean in Healthcare^34^	IHI white paper on this framework
Lean Six Sigma	Lean Six Sigma: some basic concepts^23^	Publication by NHS Institute for Innovation and Improvement on merging Lean and Six Sigma
Six Sigma	An Overview of Six Sigma^19^	Framework guidance by NHS Improvement
PRISM	A Practical, Robust, Implementation and Sustainability Model (PRISM) for Integrating Research into Practice^21^	Publication by the developers of PRISM
RE-AIM	Evaluating the Public Health Impact of Health Promotion Interventions: the RE-AIM framework^14^	Publication by the developers of RE-AIM, recommended on the official RE-AIM website.
Always Events	Always Events Toolkit^17^	IHI publication in collaboration with NHS England
FOCUS-PDSA	Introduction to Quality Improvement and the FOCUS-PDSA Model^35^	Guidance by American College of Cardiologists
Quality Implementation and Evaluation	The Quality, Implementation andEvaluation Model: A Clinical Practice Model for Sustainable Interventions^36^	Paper presenting this model.
Sustainable-QI (Sus-QI)	Sustainability in Quality Improvement: Redefining Value^16^	Guidance document recommended on the official Sus-QI website.

FOCUS-PDSAFind, Organise, Clarify, Understand, Select - Plan, Do, Study, Act NHSNational Health Service RE-AIMReach, Efficacy, Adoption, Implementation and Maintenance

After coding these documents, we categorised our findings according to the following themes:

1. Limited explicit consideration of equity or targeted QI for disadvantaged groups in QI Frameworks.
2. Indirect considerations of equity through patient involvement and targeted QI interventions.
3. There is a greater focus on reducing variation in healthcare than equity.

### Theme 1: limited explicit consideration of equity or targeted QI for disadvantaged groups in QI frameworks

There was limited explicit discussion of health equity or targeted QI in the QI Frameworks we examined. Occasionally equity was mentioned in the introductory text, in reference to the six domains of quality, but not followed through in the framework guidance itself. An example of this occurred in the IHI online guidance for the Model for Improvement. The framework advised on setting ‘equitable’ aims, in order to ‘close racial and ethnic gaps in health status’.^9^

This shows that equity is acknowledged as one of the domains of quality, however, there is no further guidance on how to implement this in practice. In this example, the definition of equitable care is limited to race and ethnicity, while equity is a much broader concept.

We found explicit consideration of health equity in two framework documents. RE-AIM, (Reach, Efficacy, Adoption, Implementation and Maintenance) a framework developed for healthcare, had the greatest consideration of equity. The framework acknowledges that projects often ‘*explicitly restrict selection of participating communities to those most motivated, organised and prepared for change’*, which can be ‘*unrepresentative of the settings to which their results are to be applied’* (Glasgow^14^, p1).

The framework also considers the importance of considering the social determinants of health when undertaking a QI project:

With the… dramatic impact of socioeconomic status on health status, understanding the degree to which a program reaches those in need is vital (Glasgow^14^, p2).

The SUS-QI framework, developed recently by the Centre for Sustainable Healthcare in partnership with the Royal College of Physicians,^15^ also shows some explicit consideration of health equity. Its key principle is to improve healthcare quality through a ‘triple bottom line’ of environmental, social and financial impacts, to ensure QI is practised sustainably. (Mortimer^16^, p2). The guidance document suggests ‘avoiding underuse of high-value interventions in people with greater need’ (Mortimer^16^, p2). This is an equity-focused principle, advocating for QI resource allocation to be distributed according to need.

There were no other explicit references to health equity in any of the coded framework documents. However, there were some indirect discussions of equity, through the lens of the social determinants of health and patient involvement.

### Theme 2: indirect considerations of equity through patient involvement and targeted QI interventions

Some frameworks do not specifically mention equity but highlight core principles that could support equity-focussed QI. For example, incorporating patient characteristics into care delivery or advocating for more patient involvement in QI. We found two ways equity was indirect considered:

#### Patient involvement

Always Events is a QI concept that has patient involvement and codesign at its core. (IHI^17^, p4). The guidance highlights the need to include a diverse group of patients:

Patients, their care partners, and service users should represent the age, race and ethnicity, or socioeconomic status in your organization.” (IHI^17^, p7)

However, the guidance does not link greater representation of marginalised groups with equity or provide recommendations about how to select a representative sample.

Another implicit reference to equity in the Always Events framework is through ‘What Matters to Patients’. For example, ‘Listen carefully and actively; be careful to listen for new information, not just to confirm pre-existing beliefs’ (IHI^17^, p11) could all encourage QI practitioners to address their own implicit biases towards certain patient groups and ideas.

The RCGP QI Wheel,^8^ Lean^18^ and Six Sigma^19^ discuss the importance of considering patient perspectives in QI. However, these references to patient involvement are not directly linked to equity and patient involvement does not guarantee equitable outcomes.^20^

#### Targeted interventions

The RCGP QI Wheel encourages primary care practitioners to consider social and demographic cultural factors in QI. It states that ‘*Success is more likely when your quality improvement intervention is appropriate for the demographics of age, gender, race, religion, and socio-economic status of the population affected.’* (RGCP^8^ p17). However, the framework does not provide guidance on how to achieve this, and how to make sure it is equitable. Similarly, PRISM (Practical, Robust Implementation and Sustainability Model) framework encourages QI teams to consider ‘*the targeted patients’ range of characteristics…such as age, gender, socioeconomic status, health literacy, native language, culture’* (Feldstein and Glasgow^21^, p10).

Always Events and Lean show some consideration of targeting resources to different patients, but there is a lack of clear guidance on how to be equitable in this decision. For example, Always Events promotes the approach of ‘*Delivering on the ‘Always’ for all patients’* (RGCP^17^, p14). But it does not discuss how to balance patients’ competing requirements or underlying inequalities. Lean, a framework developed in manufacturing but applied to healthcare, also acknowledges that ‘*all patients are different’* (NHS England^18^, p10). However, Going Lean does not advise on how to incorporate information on different patient needs into QI.

Finally, there are some indirect considerations of equity via targeted interventions in the Sus-QI framework guidance:

A culture of resource stewardship is required, ensuring that the right care is provided to the right patient at the right time to achieve the outcomes that the patient values. (Mortimer *et al*^16^, p3).

This encourages healthcare professionals to consider who is the ‘right patient’ for an intervention without directly linking to equity.

Theme 2 outlines an awareness of the importance of patient involvement and targeted interventions when doing EF-QI. However, our data also demonstrate a lack of explicit guidance in the implementation of these principles. Without explicit mentions of equity, the implementation of these principles is open to interpretation. Patient involvement is an important aspect of QI and has the capacity to reduce inequalities. But there is also the risk that QI is centred around patients with strong existing access to healthcare and those already well represented.^22^

### Theme 3: there is a greater focus on reducing variation in healthcare activity rather than equity

Many of the frameworks we analysed have a large focus on reducing variation in healthcare processes. While reducing variation in health outcomes could promote equity, there is a risk that process standardisation could impede equitable outcomes.

Lean and Six Sigma discuss how standardisation can make healthcare more efficient, through reducing variation in processes. They aim to ‘understand and reduce variation’ (NHS England^23^, p5) allowing ‘more work to be done using the same resources’ (NHS England^18^, p14).

These two frameworks view variation in healthcare as something to be reduced and standardised processes as the goal. In equity-focused QI, varying resource allocation allows it to be targeted to patient needs. The frameworks do not distinguish between variation that is needed to ensure resources are allocated proportionate to need and that which should be eliminated. For example, standardisation may limit the ability to modify care according to an individual’s social circumstances and does not consider how the social determinants of health can influence the needs of patients.

## Discussion

### Statement of principal findings

Our findings showed limited consideration of health equity within QI frameworks used in primary care. There are some indirect considerations of equity, through the lens of patient involvement and sociodemographic aspects of health and evidence that of a greater focus on efficiency than equity in the documents.

### What these findings mean

Principally, our results showed that across the frameworks we examined, there are few references to health equity. Interestingly, frameworks with more consideration of equity, such as RE-AIM and PRISM, are less commonly used in the published literature. We acknowledge that there may still be examples which are not formally published, and further primary research into this topic would be valuable.

Across all the frameworks coded, most references to inequalities are implicit and could be interpreted differently by different people based on their background. Implicit references to health inequalities are often through discussion of patient involvement. Codesign, where patients are involved in QI, promotes shared decision-making and can empower patients to take an active role in their health. However, patient involvement also runs the risk of only involving traditional privileged groups within society, rather than reaching out to those who are seldomly engaged. There is, therefore, a need for frameworks to promote equitable patient involvement.

We also found implicit references to equity through targeted QI interventions for those who need them most. However, there is a lack of explicit guidance on how to interpret this in practice. In the wider literature, disaggregated data have been identified as being key to equitable and targeted QI interventions.^3 6^ However, there is little reference to data disaggregation in the frameworks we analysed.

Two frameworks, Lean and Six Sigma, focused more on the reduction of variation than on health equity. Arguably, there is ‘an incomplete transition from the industrial origins of QI science.’^6^ Many of the QI frameworks originated in industry, with aims to reduce variation and standardise processes.

Drawing parallels between healthcare and an industrial production process can prioritise efficiency and productivity over equity. Promoting equity requires providing more resources to those with the greatest need, rather than a universalist approach of providing all patients with the same services. Lean and Six Sigma do not distinguish between variation in processes and variation in outcomes. To achieve equitable outcomes, variation in the delivery of care is required.^24^ Rather than providing the same care to everyone, QI should allocate resources proportionate to need. Equity-focussed QI (EFQI) may be more inefficient in the short term but may result in improved outcomes for all patients and long-term sustainability of healthcare systems. By making the distinction between variation which is harmful and variation which is needed for equity, QI could aim for both efficiency and equity.

### Strengths and limitations

To our knowledge, this is the first study investigating health equity considerations in primary care QI frameworks. We consulted expert opinion alongside the published and grey literature to understand the kinds of documents used commonly in primary care.

Key limitations include only looking at published frameworks, which might not reflect QI in clinical practice; it is possible we missed important unpublished documents. We have primarily focused on evidence from the UK, North America, and Australia/New Zealand and only investigated frameworks written in English. It may be that there are equity-focused frameworks in non-English-speaking regions of the world.

Given that there was limited consideration of equity in the framework guidance documents, we were unable to explore the different dimensions of equity further.

### Interpretation within the context of the wider literature

Our findings are concurrent with the wider literature; there needs to be greater consideration of equity in QI. It aligns with IHI claims that equity is the ‘forgotten aim’ of healthcare QI,^3^ lagging behind progress made on the other five aspects of quality.^1^

Health inequalities are a growing concern both for the NHS and globally. This is particularly true in the aftermath of the COVID-19 pandemic^25^ There is a greater need than ever to tackle health inequalities. Equity-focused QI could be one way of making progress in reducing inequalities.

‘All QI interventions are health equity interventions’ due to every QI project having some underlying impact on inequalities, be it positive or negative.^26^ Their recommendations for EFQI include a greater understanding of existing local disparities and greater inclusion of underrepresented populations in QI. This supports our findings in theme 2, that there is a need for more guidance on targeted QI for disadvantaged groups.

While our research found a lack of consideration of equity in QI frameworks, we did find examples of equity-focused QI projects during the initial literature search. For example, Lofters *et al* found that contacting patients in their own languages showed promise for improving cancer screening uptake.^27^ Further QI projects aimed to reduce inequalities while improving quality for all patients.^28^ There are also examples of unpublished equity-focussed QI initiatives. For example, in the UK, East London Foundation Trust website shares resources to ‘support teams to pursue equity’.^29^

### Implications for policy, practice and research

Our findings suggest that the main QI frameworks used in primary care should be revised to have more explicit equity considerations, to achieve quality in accordance with the IOM’s six aims.^1^ While not all QI projects use an explicit framework, providing health professionals with easy-to-use equity guidance is likely to promote EFQI.

From a primary care perspective, The Health Foundation found that only 23% of GP partners and 25% of salaried GPs have more than 3 hours a month of protected time for QI-related activities.^30^ Given these time constraints, we do not believe that introducing a new QI framework is likely to have a significant impact. Instead, adapting currently used frameworks to be more equity-focused may help practitioners take an equity perspective.

More research is needed to understand the extent to which existing QI projects increase or decrease inequalities. Researchers should seek to identify the guiding principles that underpin QI projects successful in addressing inequalities and be aware of the factors which may inadvertently increase inequalities.

## Conclusion

Overall, we found little consideration of equity in QI frameworks used in primary care. Where equity factors are considered, it is implicit and forms a small part of the framework. Without an explicit equity focus, QI risks exacerbating existing health inequalities. Existing QI frameworks should be modified to include an equity perspective.

## Data Availability

All data relevant to the study are included in the article or uploaded as online supplemental information.
